# Evaluation of industrial dairy waste (milk dust powder) for acetone-butanol-ethanol production by solventogenic *Clostridium* species

**DOI:** 10.1186/2193-1801-3-387

**Published:** 2014-07-28

**Authors:** Victor Ujor, Ashok Kumar Bharathidasan, Katrina Cornish, Thaddeus Chukwuemeka Ezeji

**Affiliations:** Department of Animal Sciences, The Ohio State University (OSU) and Ohio Agricultural Research and Development Center (OARDC), Wooster, USA; Department of Food, Agricultural and Biological Engineering, The Ohio State University (OSU) and Ohio Agricultural Research and Development Center (OARDC), Wooster, USA; Department of Horticulture & Crop Sciences, The Ohio State University (OSU) and Ohio Agricultural Research and Development Center (OARDC), Wooster, USA

**Keywords:** *Clostridium beijerinckii*, *Clostridium acetobutylicum*, Butanol, Food waste, Milk dust

## Abstract

Readily available inexpensive substrate with high product yield is the key to restoring acetone-butanol-ethanol (ABE) fermentation to economic competitiveness. Lactose-replete cheese whey tends to favor the production of butanol over acetone. In the current study, we investigated the fermentability of milk dust powder with high lactose content, for ABE production by *Clostridium acetobutylicum* and *Clostridium beijerinckii*. Both microorganisms produced 7.3 and 5.8 g/L of butanol respectively, with total ABE concentrations of 10.3 and 8.2 g/L, respectively. Compared to fermentation with glucose, fermentation of milk dust powder increased butanol to acetone ratio by 16% and 36% for *C. acetobutylicum* and *C. beijerinckii*, respectively. While these results demonstrate the fermentability of milk dust powder, the physico-chemical properties of milk dust powder appeared to limit sugar utilization, growth and ABE production. Further work aimed at improving the texture of milk dust powder-based medium would likely improve lactose utilization and ABE production.

## Introduction

Alternative energy-related research currently receives tremendous attention, largely in response to the rising cost of gasoline, and increased depletion of fossil fuel reserves. Consequently, interest in acetone-butanol-acetone (ABE) fermentation, which dwindled following the advent of the petrochemical industry, has been revived (Qureshi and Blaschek 2000; Qureshi and Maddox 2005; Yu et al. 2007). However, a major challenge hampering re-commercialization of the ABE process is lack of economic competitiveness, stemming in part from the absence of inexpensive, readily available, and easily fermentable substrates capable of generating high ABE yields (Qureshi and Blaschek 2000; Yu et al. 2007). Interestingly, solventogenic *Clostridium* species are capable of fermenting a wide range of carbohydrates (Ezeji and Blaschek 2008), and lignocellulosic biomass has been identified as a potential substrate for inexpensive production of ABE and other fine chemicals (Ezeji and Blaschek 2008; Zhang and Ezeji 2013). However, bioconversion of lignocellulosic biomass is currently plagued by a number of limitations, notably generation of microbial inhibitory compounds during pretreatment and hydrolysis of lignocellulose to mixed sugars (Almeida et al. 2007), and inefficient utilization of the generated mixed sugars by fermenting microorganisms due to carbon catabolite repression (Ren et al. 2010). Therefore, given the broad substrate spectrum of solventogenic *Clostridium* species (Ezeji and Blaschek 2008; Servinsky et al. 2010; Yu et al. 2007), other cheap and readily utilizable substrates, whose applications in fermentation do not require pretreatment, may prove to be more cost-effective and efficient substrates than lignocellulose.

In comparison to lignocellulose, non-lignocellulosic substrates have generally been under-investigated for bio-butanol production. Some of the non-lignocellulosic substrates investigated, thus far, for ABE production include Jerusalem artichoke extract (Maddox 1980), cassava starch (Thang et al. 2010), cheese whey (Ennis and Maddox 1989; Maddox 1980; Qureshi and Maddox 2005; Stevens et al. 1988; Welsh and Veliky 1984), apple pomace (Voget et al. 1985) and starch-based packing peanuts (Jesse et al. 2002). Among these substrates, cheese whey is the most widely researched for ABE production, mostly due to its abundance, and high biological oxygen demand (BOD), which constitutes a major disposal predicament (Maddox 1980). Although some success has been recorded with whey, its use in large scale fermentation is plagued by a number of challenges, particularly its low sugar content (5%), which often warrants an initial concentration step prior to fermentation (Maddox 1980). Although glucose is the preferred sugar for solventogenic *Clostridium* species, different researchers have shown that lactose (the sugar content of whey) is utilized by these microorganisms when supplied as the sole carbon source (Bahl et al. 1986; Maddox 1980; Yu et al. 2007). Further, lactose metabolism favors butanol production over acetone (Bahl et al. 1986; Maddox 1980), an added economic incentive in light of the current impetus for butanol production.

Against a backdrop of the challenges associated with cheese whey fermentation to ABE, there is need to evaluate inexpensive non-whey-lactose-based substrates for ABE fermentation. Milk dust powder is such a substrate. Milk dust is a blend of different milk powders left over after industrial milk packaging. Milk dust powder constitutes a considerable hazard because suspensions of milk dust at 75–1000 g/m^3^ of air can explode or self-ignite upon contact with hot surfaces (Davis et al. 2011; Ministry of Labor, New Zealand 1993). A drier with a capacity of 10 metric tons per h can generate 80.3 kg of airborne milk dust powder (Prevention of dust explosions in the food industry (2010)). The projected milk dust powder production (including skimmed and whole milk powder) in the USA in 2012 was approximately 1.02 million metric tons, which translates into 116.44 metric tons per h (United States Department of Agriculture (USDA) 2012). At present, some of the milk dust powder generated from the dairy industry is used as livestock feed; however, this enormous amount of waste (equivalent to 1.15 × 10^14^ Joules/year) has potential as a substrate for ABE fermentation. Milk dust powder constitutes predominantly of lactose, with small amounts of protein, fat and minerals. In addition, it retains all the natural properties of milk, such as color, flavor and solubility. Hence, upon mixing with water, milk dust powder resembles raw milk in appearance. In addition, milk dust does not contain urea, citric, lactic, and uric acids, found in low amounts in whey (Chatzipashali and Stamatis 2012; Foda et al. 2010; Napoli 2009), which can adversely affect fermentation efficiency.

Among solvent-producing *Clostridium* species, (including *C. saccharaoperbutylacetonicum*, *C. saccharobutylicum*, *C. tyrobutyricum*, and *C. pasterianum*), *Clostridium acetobutylicum* ATCC 824 and *Clostridium beijerinckii* NCIMB 8052 are the most characterized solventogenic *Clostridium* species and strains to date, and both have been used in the fermentation of whey, with varying outcomes (Ennis and Maddox 1989; Maddox 1980; Qureshi and Maddox 2005; Stevens et al. 1988; Welsh and Veliky 1984). However, most of these studies were centered on *C. acetobutylicum*, perhaps due to higher ABE concentrations obtained with this species relative to *C. beijerinckii*. Consequently, molecular characterization of lactose transport and utilization, and the underlying regulatory machineries in *C. acetobutylicum* ATCC 824 has been vigorously pursued (Servinsky et al. 2010; Yu et al. 2007). Albeit subject to full characterization, genomic information on *C. beijerinckii* NCIMB 8052 shows the presence of many (about 47) phosphoenol-pyruvate (PEP)-dependent phosphotransferase system (PTS) genes apparently involved in the metabolism of complex carbohydrates (Shi et al. 2010), including multiple genes whose protein products are putatively involved in lactose transport and metabolism. It is likely, therefore, that lactose metabolism in *C. beijerinckii* NCIMB 8052 is similar to *C. acetobutylicum* ATCC 824. To investigate this, we evaluated ABE production from milk dust powder by *C. beijerinckii* NCIMB 8052 and *C. acetobutylicum* ATCC 824.

## Materials and methods

### Characterization of milk dust powder

Milk dust powder used in this study was obtained from International Dairy Ingredients, Inc. (Wapakoneta, Ohio, USA). Milk dust powder was recovered from the dust collector following spray-drying of milk. Prior to fermentation, milk dust powder was subjected to a series of analyses to determine the nitrogen, ash, mineral, energy (calorific value), total solid and moisture, and total organic carbon contents (methods details below).

### Determination of ash content

The ash content of milk dust powder was analyzed according to the procedure described in the Test Methods for the Examination of Composting and Compost (TMECC 2002). Pre-weighed samples were ignited in a forced air muffle furnace (Barnstead Thermolyne 30400-Series Furnace; Model: F30428C-80) in the presence of excess air at 550°C for 2–3 h followed by cooling in a desiccator at room temperature. The resulting ash was weighed and estimated as the percentage of ash content (dry wt., w/w) in each sample.

### Determination of total solid and moisture content

Total solids and moisture content were assessed using a modified version of the TMECC method 03.09-A (2002). To prevent sugar caramelization, samples of milk dust powder were subjected to drying at 50 ± 5°C, as opposed to 70 ± 5°C recommended in the TMECC protocol (TMECC method 03.09-A 2002). Drying was allowed to proceed for 24 h or until there was no further detectable change in weight. Total solid was reported as percentage of dry solid contained in the fresh sample.

### Estimation of calorific content (energy value)

Calorific value is a measure of the fat, carbohydrate and protein content of a food material. The calorific content of milk dust powder was determined with a Bomb (combustion) calorimeter (Model: C 2000 Basic version 1, IKA). Samples (200 mg) were introduced into the decomposition unit (Model: C 5010) of the Bomb calorimeter, and incinerated in the presence of pure oxygen. The Bomb calorimeter estimates gross calorific value as the quotient of the amount of heat liberated upon total combustion and the weight of the original sample. Calorific contents were determined for triplicate samples.

### Measurement of total organic carbon (TOC)

Total organic carbon (TOC) includes biodegradable sugars, protein, and fat content of a material but does not include inorganic carbonate fractions such as calcium and magnesium carbonates. The TOC of milk dust powder was measured in accordance with the TMECC method 04.01-A (2002), using a carbon analyzer (Model Vario MAX CN, Elementar Americas). Samples were briefly subjected to combustion in an oxygen-rich atmosphere in a resistance furnace at 1,370°C. The CO_2_ produced was passed through an oxygen stream in anhydrone tubes to scrub water vapor out of the stream. The dehydrated CO_2_ stream was then channeled into an infrared detector, which generates a signal proportionate to the amount of CO_2_ detected. The resulting values are reported as percentage of TOC content (dry wt., w/w) in dried samples.

### Quantification of total nitrogen (TN) content

Total nitrogen is the sum of organic- and ammonia-derived nitrogen (nitrogen from proteins, nitrates and nitrites). The total nitrogen content of a material facilitates the determination of its carbon to nitrogen ratio (C:N). C:N ratio helps to determine the fermentability of a substrate, because nitrogen is largely essential for cell growth, while the carbon content of a substrate is critical to product yield, in this case ABE. The total nitrogen content of milk dust was determined according to the TMECC 04.02-D method (oxidation by dry combustion), by employing an automated Nitrogen analyzer (Model: Vario MAX CN, Elementar Americas). Samples (150 mg) were combusted in an oxygen-rich chamber, at a temperature of about 900°C to generate a gas stream containing CO_2_, H_2_O, and N_2_. The gas stream then was fed into a separation column, which specifically removes CO_2_ and H_2_O. The pure N_2_ was passed into a thermal conductivity detector, which generates a signal proportional to the amount of N_2_ produced. Total nitrogen is presented as percentage content (dry wt., w/w) of dried samples.

### Analysis of elemental composition of milk dust powder

Elemental composition of milk dust was measured using an inductively coupled plasma optical emission spectroscope (ICP-OES, Teledyne Leeman Labs Prodigy) following protocols described in the TMECC methods (sections 04.05, 04.06 and 04.07; 2002). Fully dried samples (1 g) were transferred into polytetrafluroethylene (PTFE) or Teflon vessels and solubilized in concentrated HNO_3_ (7 mL). Samples were microwave-digested as described in the TMECC method (section 04.12-A). When compared to alternative methods, microwave digestion allows for a more rapid digestion as it employs high pressure and temperature within the vessels and the use of closed vessel prevents cross-contamination among samples and loss of volatile elements (Sun et al. 2000). The digested samples were allowed to cool to room temperature before being transferred to an ICP-OES auto-sampler for analysis. The ICP uses argon (~10–15 L/min) to ionize the digested samples in an applied radio frequency field. Post ionization, each element exhibits a distinctive emission spectrum, of which the identity and intensity is detected and quantified by the detector (Sun et al. 2000). The concentration of each element is expressed as a function of the intensity of the corresponding elemental spectrum. The resulting concentrations are reported in mg/g sample (on a dry weight basis).

### Bacterial strains and culture conditions

*C. beijerinckii* NCIMB 8052 (*C. beijerinckii* ATCC 51743; hereafter referred to as *C. beijerinckii*) and *C. acetobutylicum* ATCC 824 (hereafter referred to as *C. acetobutylicum*) obtained from the American Type Culture Collection (Manassas, VA) were used in the fermentation of milk dust powder. Spores were stored in sterile, double-distilled water at 4°C. To revive spores for inoculation, stocks (200 μL) were heat-shocked for 10 min at 75°C followed by cooling on ice. The heat-shocked spores were then inoculated into anoxic pre-sterilized tryptone–glucose–yeast extract (TGY) broth (10 ml) and incubated in an anaerobic chamber (Coy Laboratory Products Inc., Ann Arbor, Michigan), with a modified atmosphere of 82% N_2_, 15% CO_2_, and 3% H_2_ for 12 h to 14 h at 35°C ± 1°C. When the optical density (OD_600nm_) reached 0.9–1.1, 8 ml of actively growing culture was transferred into 92 mL of anoxic TGY medium and incubated as above until the OD_600nm_ reached of 0.9 - 1.1 (Zhang and Ezeji 2013; Han et al. 2011). This was used as the pre-culture to inoculate the milk dust-based fermentations.

Batch ABE fermentation by *C. beijerinckii* and *C. acetobutylicum* was performed in 150 ml Pyrex screw-capped media bottles containing anoxic milk dust-based medium (in a final volume of 100). The milk dust powder medium was prepared by autoclaving a mixture of ~12 g milk dust powder and 1g yeast extract at 121°C for 15 min. Upon cooling to 40°C, the mixture was transferred into the anaerobic chamber and ~80 ml anoxic, sterilized distilled water was added to bring the final concentration of lactose in the medium to 50 g/L. Prior to inoculation, 1ml each of filter-sterilized P2 stocks including vitamin (0.1 g/L *para*-amino-benzoic acid; 0.1 g/L thiamine; 0.001 g/L biotin), buffer (50 g/L KH_2_PO_4_; 50 g/L K_2_HPO_4_; 220 g/L ammonium acetate) and mineral (20 g/L MgSO_4_.7H_2_O; 1 g/L MnSO_4._H_2_O; 1 g/L FeSO_4_.7H_2_O; 1 g/L NaCl) solutions were added (Richmond et al. 2012; Zhang et al. 2012). As a control, 100 ml of P2 medium (glucose, 60 g/L and yeast extract, 1 g/L) containing P2 stock solutions was inoculated with both *C. acetobutylicum* and *C. beijerinckii*. All cultures were inoculated with 6% pre-culture for both species studied. Samples were taken every 12 h for pH, residual sugars, ABE, and acid analyses. Unless otherwise stated, all fermentations were conducted in triplicate at 35 ± 1°C, and no agitation or pH control was employed.

### Analytical procedures

Owing to the cloudiness of the milk dust medium, bacterial growth was determined by plate count (viable cell count). Each colony forming unit (CFU) was regarded to have originated from a single cell. Culture samples were serially diluted in 10 ml of TGY medium and 100 μL of serially diluted samples were plated on 10 ml semi-solid TGY agar (0.45% agar in TGY medium). The plates were incubated anaerobically for 24–48 h at 35 ± 1°C and the number of CFUs was counted and expressed as CFU per ml of original culture (Jesse et al. 2002; Nielsen et al. 2009). Concentrations of glucose and lactose were measured by high performance liquid chromatography (HPLC) with a refractive index (RI) detector (Agilent Technologies 1200 Series) using an organic acid column (Rezex ROA-Organic Acid H^+^ column, 300 mm × 7.8 mm). The mobile phase was 0.0025 M H_2_SO_4_ (Fluka) operated at a flow rate of 0.6 ml/min. All samples were injected by automatic sampler and the injection volume was 10 μL. The column and detector temperature were maintained at 80°C and 55°C respectively.

The pH profiles of fermentation cultures were monitored with a Beckman Ф500 pH meter (Beckman Coulter Inc., Brea, CA). Concentrations of fermentation products, namely, acetate, butyrate, acetone, butanol, and ethanol, were measured using a 7890A Agilent Technologies gas chromatograph (Agilent Technologies Inc., Wilmington, DE) equipped with a flame ionization detector (FID) and 30 m (length) × 320 μm (internal diameter) × 0.50 μm (HP-Innowax film) J x W 19091N-213 capillary column as described previously (Han et al. 2011, 2012), with nitrogen as carrier gas. The inlet and detector temperatures were maintained at 250°C and 300°C respectively. The temperature of the oven was programmed from 60–200°C with 20°C/min increments, and a 5-min hold at 200°C. One microliter was injected per sample with a split ratio of 10:1. Yield was calculated as the maximum amount of butanol/ABE produced per gram of substrate utilized (expressed in g/g substrate). ABE productivity was estimated as maximum ABE produced (g/L) divided by the corresponding fermentation time in hours (Ezeji and Blaschek 2008).

## Results

### The physico-chemical characteristics and composition of milk dust powder

HPLC analysis showed that the milk dust powder used in this study had lactose content of ~425 g/L (w/v; wet wt.); 8.5-fold higher than cheese whey, which typically has a lactose content of about 50 g/L (Welsh and Veliky 1984). The total solid and calorific contents, and the carbon:nitrogen ratio of the milk dust powder were ~94%, 50 kJ/kg and 7, respectively (Table 1). The predominating elements were potassium (K), phosphorus (P), and calcium (Ca) with concentrations of 13.05, 6.38, and 7.08 mg/g dry matter, respectively (Table 1). Copper (Cu) was the least element present with a concentration of 0.0004 mg/g dry matter (Table 1).

**Table 1:** **The physico chemical properties of milk dust powder**

% Ash	% Total solids	Calorific value (kJ/kg)	% Carbon	% Nitrogen	C/N ratio	Elemental composition (mg/g dry matter)												
						P	K	Ca	Mg	S	Al	B	Cu	Fe	Mn	Mo	Na	Zn
3.79 ± 0.21	93.64 ± 0.10	50.20 ± 1.92	45.51 ± 0.30	6.46 ± 0.10	7.04 ± 0.33	6.38 ± 0.15	13.05 ± 0.31	7.08 ± 0.25	0.89 ± 0.15	4.02 ± 0.33	0.03 ± 0.11	0.01 ± 0.12	0.0004 ± 0.22	0.03 ± 0.36	0.0002 ± 0.08	0.001 ± 0.05	4.03 ± 0.38	0.03 ± 0.05

### Growth profiles of *C. acetobutylicum*and *C. beijerinckii*on milk dust medium

To assess the growth of *C. acetobutylicum* and *C. beijerinckii* on milk dust medium, the growth profiles of both species on milk dust medium were compared against each other and in relation to control fermentations on glucose, their preferred substrate. Microbial cell counts showed that both *C. acetobutylicum* and *C. beijerinckii* grew better on glucose than on lactose (milk dust) (Table 2). Maximum cell count obtained with glucose-grown cultures of *C. acetobutylicum* (1.39 × 10^11^) was 18-fold higher than the maximum count for cultures grown on milk dust medium (7.67 × 10^9^). The growth of *C. beijerinckii* on glucose (1.1 × 10^10^) was only 3-fold better than on milk dust medium (3.67 × 10^9^). Compared to *C. beijerinckii*, the colony-forming units of *C. acetobutylicum* were 2.1-fold higher, when both microorganisms were grown on milk dust powder. It is noteworthy, however, that the improved growth of *C. acetobutylicum* over *C. beijerinckii* was more pronounced when both were grown on glucose, in which the cell count for *C. acetobutylicum* was ~13-fold higher than that obtained with *C. beijerinckii*.

**Table 2:** **Growth (colony-forming units/ml) of** ***C. acetobutylicum*** **and** ***C. beijerinckii*** **on milk dust powder based medium**

Time (h)	Glucose***C. acetobutylicum***ATCC 824 cfu/ml	Milk dust***C. acetobutylicum***ATCC 824 cfu/ml	Glucose***C. beijerinckii***NCIMB 8052 cfu/ml	Milk dust***C. beijerinckii***NCIMB 8052 cfu/ml
0	2.86 × 10^7^	1.87 × 10^7^	5.1 × 10^7^	1.17 × 10^7^
12	8.06 × 10^9^	3.1 × 10^8^	2.0 × 10^9^	1.27 × 10^8^
24	4.46 × 10^10^	4.0 × 10^9^	3.3 × 10^9^	4.67 × 10^8^
36	1.39 × 10^11^	7.67 × 10^9^	1.1 × 10^10^	3.67 × 10^9^
48	8.3 × 10^10^	1.03 × 10^9^	8.6 × 10^9^	2.33 × 10^9^
60	6.1 × 10^10^	1.13 × 10^9^	6 × 10^9^	1.3 × 10^8^
72	6.9 × 10^9^	7.0 × 10^8^	1.2 × 10^9^	1.6 × 10^8^

### Fermentation profiles of *C. acetobutylicum*and *C. beijerinckii*

**Figure 1:**
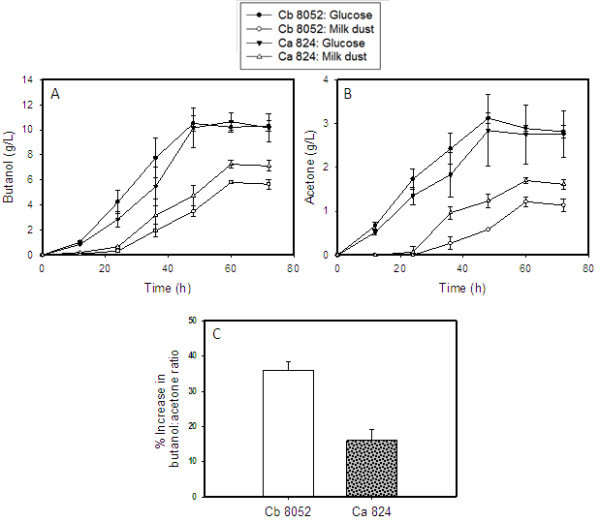
**The concentrations of butanol , and acetone of** ***C. acetobutylicum*** **and** ***C. beijerinckii*** **on milk dust powder, and their butanol:acetone ratios relative to glucose grown cultures. (A)** Butanol produced during ABE fermentation, **(B)** acetone produced during ABE fermentation, and **(C)** increase in butanol:acetone ratio in cultures grown in milk dust powder medium relative to cultures grown in glucose medium. Ca 824: *C. acetobutylicum*; Cb 8052: *C. beijerinckii*.

**Figure 2:**
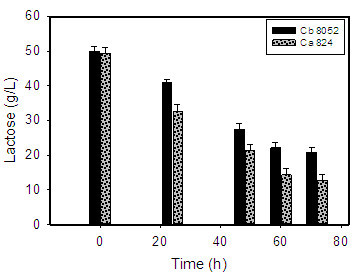
**Lactose utilization profiles of** ***C. acetobutylicum*** **and** ***C. beijerinckii*** **.** Ca 824: *C. acetobutylicum*; Cb 8052: *C. beijerinckii*.

As with growth, both microorganisms performed better on glucose than in milk dust-based medium. *C. acetobutylicum* produced more ABE than *C. beijerinckii* when both were grown in milk dust medium (Table 3, Figure 1). Cultures of *C. acetobutylicum* grown in milk dust medium produced 7.3 and 10.3 g/L of butanol and ABE, respectively. Both were 1.3-fold higher than the butanol (5.8 g/L) and ABE (8.2 g/L) concentrations produced by *C. beijerinckii* in milk dust medium (Table 3, Figure 1A). However, a preponderance of butanol over acetone in cultures grown on lactose as the main carbon source (Maddox 1980) was observed with both species. Whereas *C. acetobutylicum* produced 1.7 g/L acetone, *C. beijerinckii* produced 1.2 g/L (Figure 1B) when grown in milk dust medium. As a result, butanol:acetone ratios for both microorganisms were higher on milk dust relative to glucose. In fact, the ratios of butanol to acetone in cultures of *C. beijerinckii* and *C. acetobutylicum* grown on milk dust powder were 36% and 16% higher, respectively, relative to glucose-grown cultures (Figure 1B). This is over 2-fold higher with *C. beijerinckii* when compared to *C. acetobutylicum*. The ABE yield of *C. acetobutylicum* did not vary significantly from that of *C. beijerinckii* despite producing a higher ABE concentration (Table 3). This is ascribable to higher lactose consumption by *C. acetobutylicum*, which utilized 34.7 g/L of lactose; 30% higher than the 27.7 g/L consumed by *C. beijerinckii* (Figure 2).

**Table 3:** **Sugar utilization, ABE concentrations, yields and productivities of** ***C. acetobutylicum*** **and** ***C. beijerinckii***

Parameters	Glucose***C. acetobutylicum***ATCC 824	Milk dust***C. acetobutylicum***ATCC 824	Glucose***C. beijerinckii***NCIMB 8052	Milk dust***C. beijerinckii***NCIMB 8052
Acetone (g/L)	2.84 ± 0.82	1.70 ± 0.07	3.12 ± 0.12	1.21 ± 0.11
Ethanol (g/L)	1.39 ± 0.33	1.45 ± 0.21	1.33 ± 0.48	1.16 ± 0.21
Butanol (g/L)	**10.65 ± 0.69**	**7.25 ± 0.32**	**10.51 ± 0.65**	**5.80 ± 0.12**
Total ABE (g/L)	**14.62 ± 1.74**	**10.25 ± 0.62**	**14.97 ± 1.25**	**8.15 ± 0.79**
Initial glucose/lactose (g/L)	60.89 ± 0.16	49.94 ± 1.61	60.86 ± 0.37	49.08 ± 1.26
Final glucose/lactose (g/L)	**20.44 ± 1.92**	**14.36 ± 1.66**	**20.24 ± 2.44**	**22.26 ± 1.43**
Total glucose/lactose utilized (g/L)	40.45 ± 1.77	34.72 ± 1.64	40.62 ± 2.08	27.67 ± 0.70
ABE yield (g/g of substrate)	0.36 ± 0.01	0.30 ± 0.03	0.37 ± 0.02	0.29 ± 0.02
ABE productivity (g/L/h)	0.25 ± 0.01	0.17 ± 0.01	0.31 ± 0.02	0.13 ± 0.01

**Figure 3:**
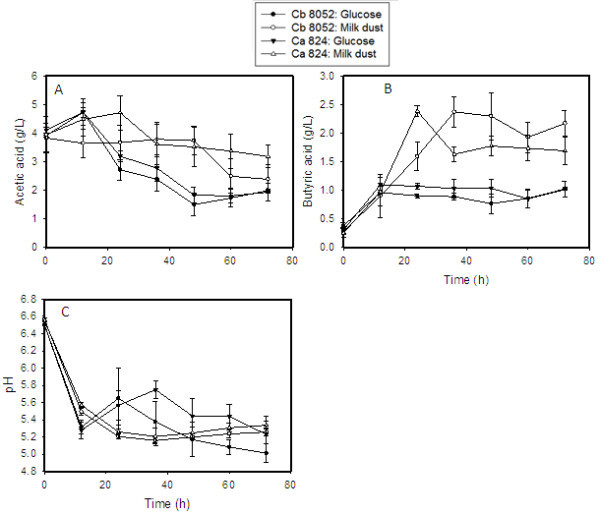
**The levels of acetate and butyrate and pH profiles of cultures of** ***C. acetobutylicum*** **and** ***C. beijerinckii*** **grown on milk powder medium. (A)** Acetic acid concentration profile during ABE fermentation, **(B)** butyric acid concentration profile during ABE fermentation, and **(C)** pH profile during ABE fermentation. Ca 824: *C. acetobutylicum*; Cb 8052: *C. beijerinckii*.

Notably, acetate and butyrate levels in cultures grown in milk dust medium were considerably higher than the levels detected in cultures grown on glucose (Figure 3). Both fermentation media (glucose- and milk dust-based) contain acetate (2.2 g/L) in addition to acetate carried over from the preculture. As a result, acetate levels at 0 h were ~4 g/L. In glucose-based medium, acetate levels dropped sharply, while they either increased or remained relatively stable in cultures grown on milk dust. Consequently, over the course of fermentation, acetate levels in milk dust media were 1.2- to 2.5-fold higher in cultures grown on milk dust when compared to those grown on glucose, over the course of fermentation (Figure 3). Similarly, butyrate levels were considerably higher in milk dust medium than in the glucose medium (1.3- to 2.5-fold) for both *C. acetobutylicum* and *C. beijerinckii*. Despite the differences in acetate and butyrate levels between cultures grown on glucose and those grown on lactose (milk powder-based medium), the pH profiles of both sets of cultures varied only slightly (Figure 3B). Variations in pH were more obvious with *C. acetobutylicum* than with *C. beijerinckii*. With *C. beijerinckii*, the pH values were slightly higher in the glucose medium than the milk dust powder medium only at 24 and 36 h. Conversely, with *C. acetobutylicum*, pH remained marginally higher in the glucose-grown cultures than those grown on milk dust from 24 to 60 h.

## Discussion

Return of ABE fermentation to industrial relevance is contingent upon the availability of readily fermentable inexpensive substrate(s), with high product yield (Yu et al. 2007). Our results demonstrate that milk dust powder can be fermented to butanol without the usual concentration, pH adjustment, and deproteination processes (Welsh and Veliky 1984) often associated with cheese whey fermentation. Further, our results substantiate previous reports (Bahl et al. 1986; Maddox 1980) that lactose fermentation favors butanol production over acetone, as both *C. acetobutylicum* and *C. beijerinckii;* particularly the latter showed higher butanol:acetone ratios on milk dust powder relative to glucose.

**Figure 4:**
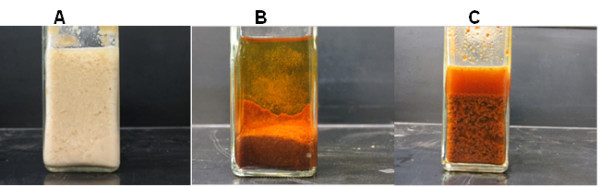
**Physical properties of milk dust medium used for ABE fermentation by** ***C. acetobutylicum*** **and** ***C. beijerinckii*** **. (A)** Coagulated milk dust powder prior to autoclaving, **(B)** coagulated milk dust powder after autoclaving, and **(C)** coagulated milk dust powder medium was shaken prior to inoculation with *C. acetobutylicum* or *C. beijerinckii.*

When compared to the glucose medium, a number of factors may account for the lower product concentrations and yields on milk dust medium (Table 2). First, the preference of glucose over lactose by solventogenic clostridia may be a dominant factor underlying this pattern. Secondly, the physico-chemical properties of milk dust powder may have negatively influenced growth and ABE production, as cultures grown on milk dust-based medium exhibited a prolonged lag phase, resulting in delayed ABE production (Figure 1). Whereas accumulation of ABE increased significantly 12 h after inoculation in the glucose medium, a substantial increase in ABE production was not observed in the milk powder medium until 24 h post inoculation (Figure 1). After heat-sterilization of milk powder, reconstitution in water resulted in the formation of hard, crystal-like balls, which reduced the medium surface area, thereby limiting sugar uptake. Milk dust powder constitutes largely of casein (~80%) and whey (20%) proteins (Farell et al. 2004). When milk dust is subjected to elevated temperatures (90°C–120°C for 10 or more min), casein and whey undergo coagulation and denaturation, respectively (Cleaning and Sanitizing of Containers and Equipment (2005); Coagulation Of Milk, Part 3 (2007); Bender 2005). This is because the stability of casein in milk dust during heat treatment is dependent on the availability of optimal amounts of calcium and magnesium (present in phosphate and citrate forms) which limit the alignment of casein into a three-dimensional lattice that holds sugars, fats, and water in position thus preventing their availability for microbial uptake. Although the milk dust powder used in this study was found to contain significant levels of calcium, magnesium and phosphorus (Table 1), heat treatment affects salt equilibria leading to the precipitation of calcium and magnesium phosphates/citrates (http://drinc.ucdavis.edu/dairyp/dairyp5.htm; http://www.chestofbooks.com/food/science/Experimental-Cookery/Coagulation-Of-Milk-Part-3.html; Bender 2005). Consequently, this aggravates casein coagulation, and the resulting crystals drastically reduce the availability of sugars to the fermenting cells. Physically breaking these hard crystals were found to enhance growth and ABE production (Figure 4C). In addition to influencing lactose utilization and ABE production, it is likely that these physical characteristics affected analytical measurement of lactose in solution given that the milk dust powder did not dissolve completely in water.

Thirdly, ABE fermentation is pH-sensitive because of the delicate interplay between fermenting cells and acids (acetate and butyrate), relative to ABE production (Grupe and Gottschalk 1992). However, we have observed that pH values within 5.2 and 5.8 favor ABE productions. Although the pH ranges of both microorganisms investigated in this study were well within this range during growth in milk dust medium, ABE production was nonetheless less than the levels observed with glucose. In view of this, we contend that the pH values might have been influenced by the heterogeneous nature of the milk dust powder medium (Figure 4), as the pH values do not mirror the corresponding acid levels. With the cloudy consistency of milk dust powder coupled with the presence of large particles, it is likely that pockets of microbial activity and inactivity or low activity may be distributed in the culture. In addition, the pH of ABE fermentation broth is significantly influenced by the ratio of protonated acetic and butyric acids to their unprotonated forms (Farell et al. 2004; Bryant and Blaschek 1988; Russell and Diez-Gonzalez 1998; Ezeji et al. 2010). Given the higher acid levels detected in cultures of *C. acetobutylicum* and *C. beijerinckii* grown on milk dust powder medium, relative to cultures grown on glucose, it may be deduced that acid reassimilation was impaired in the milk dust powder medium (Figure 3A and B). Poor acid reassimilation has been reported for substrate-limited cultures of solventogenic clostridia (Jesse et al. 2002). Reduced availability of lactose in the milk dust medium owing to coagulation of casein post sterilization is therefore a probable factor contributing to the higher acetic and butyric acid levels detected in this medium. Although reduced nutrient uptake should affect acid production pre-solventogenesis, the produced acids are less readily reabsorbed with nutrient limitation (Jesse et al. 2002), and the media used in this study (both glucose- and milk dust powder-based) contained 2.2 g/L acetic acid from the onset of fermentation. Taken together, these factors would ultimately dampen ABE production.

Clearly, *C. acetobutylicum* performed better than *C. beijerinckii* on milk dust medium with respect to growth (Table 2), lactose utilization (Figure 2) and ABE production (Table 3; Figure 1), but not on glucose. This may be indicative of superior lactose transport and metabolism by *C. acetobutylicum* relative *C. beijerinckii. C. acetobutylicum* has been shown to possess robust lactose metabolic machinery (Servinsky et al. 2010). On the other hand, there is a paucity of experimental data on the utilization of lactose by *C. beijerinckii*. Hence, studies targeted at unraveling the regulatory machineries that govern lactose utilization in *C. beijerinckii* may prove instructive. We anticipate that the findings of this study would encourage such investigation with a view to delineating the discrepancy in lactose utilization, and consequently, ABE production between *C. acetobutylicum* and *C. beijerinckii* on a lactose-replete medium.

This study demonstrates for the first time, fermentation of non-whey, lactose-rich industrial diary waste for ABE production. Fermentation of milk dust powder by both species studied favors butanol production over acetone, similar to previous reports for whey. Taken together, availability of cheap milk dust powder, calls for further investigation towards improving its fermentability for enhanced ABE production.
